# Investigation on Rheological Properties of Water-Based Novel Ternary Hybrid Nanofluids Using Experimental and Taguchi Method

**DOI:** 10.3390/ma15010028

**Published:** 2021-12-21

**Authors:** Jalal Mohammed Zayan, Abdul Khaliq Rasheed, Akbar John, Mohammad Khalid, Ahmad Faris Ismail, Abdul Aabid, Muneer Baig

**Affiliations:** 1 Department of Mechanical Engineering, Faculty of Engineering, International Islamic University Malaysia, Kuala Lumpur 50728, Selangor, Malaysia; zayan_mohammed@yahoo.co.in (J.M.Z.); faris@iium.edu.my (A.F.I.); 2 Department of New Energy Science and Engineering, School of Energy and Chemical Engineering, Xiamen University Malaysia, Jalan Sunsuria, Bandar Sunsuria, Sepang 43900, Selangor, Malaysia; 3 Institute of Oceanography and Maritime Studies (INOCEM), International Islamic University Malaysia, Kuantan 25200, Pahang, Malaysia; 4 Graphene & Advanced 2D Materials Research Group (GAMRG), School of Engineering and Technology, Sunway University, No. 5, Jalan Universiti, Bandar Sunway, Petaling Jaya 47500, Selangor, Malaysia; khalids@sunway.edu.my; 5 Engineering Management Department, College of Engineering, Prince Sultan University, P.O. Box 66833, Riyadh 11586, Saudi Arabia; mbaig@psu.edu.sa

**Keywords:** ternary hybrid nanoparticles, hybrid nanofluids, shear rate, Taguchi method, heat transfer

## Abstract

This study presents the rheological behavior of water-based GO-TiO_2_-Ag and rGO-TiO_2_-Ag ternary-hybrid nanofluids. The impact of nanoparticles’ volumetric concentration and temperature on the rheological properties were studied. All experiments were performed under temperatures ranging from 25 to 50 °C in the solid volume concentration range of 0.5–0.00005%. The data optimization technique was adopted using the Taguchi method. The types of nanomaterials, concentration, temperature, and shear rate were chosen to optimize the viscosity and shear stress. The effect of shear stress, angular sweep, frequency sweep, and damping factor ratio is plotted. The experimental results demonstrated that the rheological properties of the ternary hybrid nanofluid depend on the ternary hybrid nanofluid’s temperature. The viscosity of ternary hybrid nanofluids (THNf) change by 40% for GO-TiO_2_-Ag and 33% for rGO-TiO_2_-Ag when temperature and shear rates are increased. All the ternary hybrid nanofluids demonstrated non-Newtonian behavior at lower concentrations and higher shear stress, suggesting a potential influence of nanoparticle aggregation on the viscosity. The dynamic viscosity of ternary hybrid nanofluid increased with enhancing solid particles’ volume concentration and temperature.

## 1. Introduction

The concept of “nanofluid” was first coined by Choi [1] to describe the process of making stable colloidal suspensions of solid nano-sized particles (1–100 nm) in common base fluids. Nanofluids were proposed as novel heat transfer fluids as they can significantly enhance conventional liquids’ thermophysical and rheological characteristics. Nanoparticles are typically dispersed in base fluids, such as water, ethylene glycol, oils, and synthetic fluids, such as liquid paraffin oil and polyester. Gold, silver, titanium, aluminum, iron oxide, etc., are commonly used metallic nanoparticles. The carbon-based nanoparticles used are diamonds, graphene, CNT, fullerenes, etc. Some other non-carbon, non-metallic nanoparticles include sodium, ceramic, carbides, etc. [2]. They are adapted in many major industrial and biomedical applications where rapid heating and cooling are critical, such as engines, power plants, pharmaceutical processes, vehicle thermal management, to name a few [3]. As a result, nanofluids have garnered considerable interest owing to their potential benefits in a wide variety of applications. Substantial research has been done on the rheological properties of fluids which deals with the study of their viscosity or their resistance to deformation and thermophysical properties, fluid flow, and heat transfer [4,5]. Rheology is a correlation of stress and strains that can study and understand fluids’ fundamental behavior and characteristics, which are essential for deriving viscosity models. Dispersing nanoparticles in base fluids increases the thermal conductivity and alters the fluids’ rheological behavior and other structural properties. The detailed rheological analysis of nanofluids will enable us to determine the fluid’s Newtonian or non-Newtonian nature, the structural evolution of solid-liquid dispersion, and pumping power, and interpret and quantify the mechanics of the solid-fluid interaction under various temperature and stress conditions. The stability of the nanoparticle in fluid and its duration without sedimentation, etc., also influence the viscosity of nanofluids. Because of all these considerations, it is crucial to study the effect of dispersing nanoparticles in base fluids on the structure of fluids and their rheological behavior, change in their chemical structure under different stress and temperature conditions [6]. Studies have investigated the effects of nanoparticles on the fluid-structure and also the type of nanoparticles used, such as graphene [7], gold [8], aluminum, hybrid nanoparticles [6], and other metallic/non-metallic/carbon nanoparticles-based nanofluids and their applications [9]. Previous research has shown an increase in thermal conductivity, which has enhanced the efficiency of their applications. One such research using nanoparticles was shown to accelerate thermal conductivity of polymerase chain reaction (PCR) by significantly reducing the number of cycles with the addition of graphene nanoflakes [10] and titanium nanoparticles [11]. Few other studies have observed that with the addition of nanoparticles, the applications yielded better outcomes in fields such as medical [8], nuclear reactors [12], oil recovery [13], etc.

Hybrid nanoparticles are a combination of two or more nanoparticles synthesized or decorated on top of each other, and their dispersion into base fluids is known as hybrid nanofluids. They have a unique attribute of improved thermal conductivity and tailor-made rheological properties that make them desirable for various applications. These properties of nanofluids have attracted many researchers to explore the applications of hybrid nanofluids in diverse fields of applications [14]. Numerous studies and reviews have been published on hybrid nanofluid preparation methods [15], their characterization [16], thermal conductivity enhancements [17], thermal conductivity models [18], thermal conductivity mechanisms [19], the effect of particle size and shape on thermal conductivity [20], the rheological effects of hybrid nanoparticles dispersion on-base fluids [6], rheological behavior [21], viscosity [22], viscosity models [23], the effect of particle size and shape on the viscosity [24], and stability [2]. Studies on hybrid nanofluids have gained momentum only recently [9]. Bi-hybrid nanoparticles were used in various studies, such as CuO-MWCNT [25], Al_2_O_3_-TiO_2_ [26], SiO_2_-MWCNT [27], MWCNT-GNPs [28], to name a few. However, very few studies have been performed to date on the rheological behavior of trihybrid/ternary hybrid nanoparticles with different concentrations and their sweep characteristics, such as amplitude sweep and frequency sweep, focused on storage and loss moduli. Furthermore, relatively few studies observed the rheological effects of dispersing minute or very low concentrations of nanoparticles on base fluids (<1% wt.), which can be categorized as dilute [29]. Numerous hybrid nanofluids have been studied in the literature, including a dispersion of hybrid nanoparticles in the metallic form, such as silver-gold hybrid [30], or a mixture of carbon-metallic nanoparticles, such as graphene–titanium dioxide [31], etc., to mention a couple of them. Each of these hybrid nanoparticles has distinct characteristics and behaviors. Hybrid nanofluids have also been explored from both a theoretical and practical engineering aspect due to their superior heat transfer performance [30,31]. The use of nanofluids might be beneficial for improving heat transfer; however, the presence of nanoparticles may also increase viscosity, which may lead to higher energy consumption in terms of pumping power [32]. Hence, adding the nanomaterials as a hybrid could enhance the thermal performance and reduce the substantial volume suspended in the base liquid. It is widely known that viscosity has a more significant impact on power consumption in the laminar flow regime (ΔP = 32ηVLD^−2^) than in the turbulent flow regime (ΔP = 0.1582Lη^0.25^ρ^0.75^D^−1.25^V^1.75^). Thus, a fundamental understanding of nanofluids’ rheological behavior is gradually evolving, and attempts to perform more tests are likely to contribute towards establishing nanofluids as a viable choice for hydraulic transport and other practical uses. Previous researchers focused on the rheological properties of hybrid nanofluids, mainly of carbon nanotube [27,28], and titanium dioxide-based biohybrid nanofluid [26]. These reports demonstrate that hybrid nanofluids’ rheological properties would be influenced by flow parameters, such as solid volume fraction, temperature, shear rate, etc. Researchers have also studied the forecast of the thermal conductivity ratio of an Al_2_O_3_/water nanofluid as a function of particle size, temperature, volumetric concentration, LSSVM, and an artificial neural network [33]. Another factor to consider in this analysis is the absence of appropriate rheological observations and models from studies on tri- or ternary hybrid nanoparticles dispersed in DI water. Few researchers have used Taguchi analysis to study the grey relational analysis which is utilized to optimize the input parameters [34], viscosity and relative viscosity [35], signal to noise ratio, SNR [36], impact of concentration of nanoparticles on the fluids [37], etc. Indeed, the data optimization techniques have been found in different mechanical engineering fields, such as materials science [38,39], solid mechanics [40], and fluid dynamics [41,42,43], and claimed that they are helpful to predict the optimum result with the cost-effective and energy-saving method.

This study evaluated the time- and temperature-dependent flow behaviors of two novel ternary hybrid nanofluids, GO-TiO_2_-Ag and rGO-TiO_2_-Ag, with low solid volume fractions. Three distinct nanoparticles, namely, graphene oxide (GO), titanium oxide (TiO_2_), and silver (Ag), are layered on top of each other, forming two novel trihybrid or ternary hybrid nanoparticles (THNp), GO-TiO_2_-Ag and rGO-TiO_2_-Ag. Using the two-step method, two-hybrid water-based nanofluids were produced by dispersing the two forms of graphene-based hybrid nanoparticles in water [44]. Moreover, the continuous rheological properties’ evaluation and time/temperature-dependent rheological behaviors’ assessment of GO-TiO_2_-Ag/water nanofluids and rGO-TiO_2_-Ag/water nanofluids were at a concentration of 0.5%, 0.05%, 0.005%, 0.0005%, and 0.00005% and temperatures ranging from 25 to 50 °C. The primary reason for dilute solutions seldom being assessed or reported is that it is difficult to measure their rheological behavior. Furthermore, according to our knowledge, no study has analyzed the very complex fluids’ storage and loss moduli by measuring the hybrid nanofluids’ sweep characteristics (amplitude sweep and frequency sweep). Lastly, Taguchi design was used to study the impact of the type of nanofluid, their concentration, temperature, and shear rate on viscosity and shear stress. Nanofluid concentration was optimized to provide the optimum rheological characteristics.

## 2. Experimental Investigation

### 2.1. Synthesis of Ternary Hybrid Nanoparticles

GO-TiO_2_-Ag and rGO-TiO_2_-Ag nanocomposites were prepared by the hydrothermal approach based on our previous research [45]. Briefly, 0.25 g of GO was dispersed in 250 mL of deionized water by ultrasonic stirring for approximately 2 h. Separately, 10 mL of titanium isopropoxide (TTIP) and silver was combined with 10 mL of isopropyl alcohol, and this solution was added to 50 mL of GO suspension. After that, 10 mL of 0.2 M AgNO_3_ was added dropwise to the solution. The solution was then mixed and stirred for 30 min, and the pH was altered to 1.1 while stirring was continued for about 2 h to produce a uniform homogeneous solution. This homogeneous solution was heated at 160 °C for 24 h in a Teflon-lined stainless-steel autoclave. Next, the substance was vigorously sprayed with ethanol and then washed with water to remove all traces and unreacted ions. The GO-TiO_2_-Ag nanocomposites were obtained, the solution was purified and dried at 80 °C. The composition of GO/rGO:TiO_2_:Ag was roughly estimated to be about 5:1:1 parts by weight. The main difference between the rGO-TiO_2_-Ag and the rGO-TiO_2_-Ag nanocomposites was the addition of ammonia and hydrazine. These compounds were used to remove oxygen molecules from GO sheets as well as their functional groups. The detailed characterizations are presented in our previous work [45].

### 2.2. Preparation of Nanofluids

Before rheological testing of nanofluids there is an essential step to prepare nanofluids with good suspension stability to ensure that the nanofluids are free of sedimentation and agglomeration in the test process. In this experiment, GO-TiO_2_-Ag/water suspension and rGO-TiO_2_-Ag/water suspension at a weight concentration of 0.5% containing nanoparticles with an average diameter of 750 nm and 1750 nm, respectively, were evaluated for their stability by measuring their zeta potential using an Anton Paar Electrokinetic Analyzer SurPASS 3.

The preparation of nanofluids has a vital role in obtaining the desired stability characteristics. Improving uniform dispersal and achieving the desired properties allows a nanofluid to be durable without sedimentation. Water-based nanofluids were prepared using nanoparticles at different concentration ratios. Briefly, GO-TiO_2_-Ag THNp were suspended in deionized water at a concentration of 0.5 wt%. The nanofluids were then sonicated using an ultrasonic probe sonication for 1 min and then followed by water bath sonication for roughly 4 h to acquire a homogenous nanofluid solution without sedimentation. The period of 1-min probe ultrasonication and 4 h of water bath sonication was chosen based on early trials and subsequent iterations to improve the dispersion of decorated ternary hybrid nanoparticles in the base fluids without breaking them. The stock solution was then serially diluted at five more levels named concentration A (5 × 10^−1^ wt%), B (5 × 10^−2^ wt%), C (5 × 10^−3^ wt%), D (5 × 10^−4^ wt%), and E (5 × 10^−5^ wt%) (from semi-dilute to dilute concentrations of nanofluids).

### 2.3. Stability of Ternary Hybrid Nanofluids

**Figure 1:**
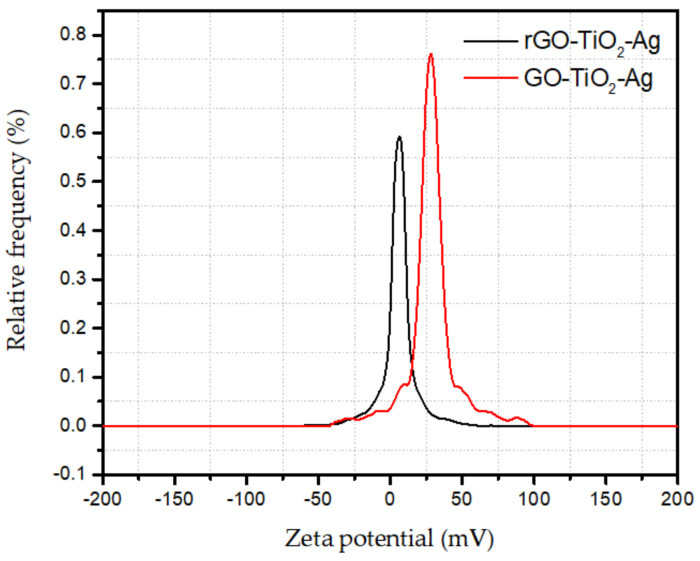
Zeta potential of ternary hybrid nanofluids.

The zeta potential of the prepared nanofluids measures the stability and surface load at a solid-liquid interface. The electrostatic repulsion forces and the zeta potential are directly proportional. The electrostatic repulsion force is supposed to be stronger than the precipitation forces of attraction. For the stabilization of nanofluids with solid dispersion, the optimal absolute zeta potential should be approximately ±30 mV. From Figure 1 it can be observed that the zeta potential of the rGO-TiO_2_-Ag-based nanofluids peak was at 32 mV, which is within a stable limit. In contrast, the GO-TiO_2_-Ag-based nanofluids peak was at 21 mV.

### 2.4. Viscosity Measurements

An Anton Paar’s modular compact rheometer (GmbH, MCR302, Austria) equipped with a Peltier balancing cell C-PTD200 palter temperature control system was used to evaluate the dynamic viscosity of hybrid nanofluids. Viscosity, shear rate, and shear stress measurements were conducted using a 1–100 1/s spindle [46]. CC45 DIN was used at temperatures ranging from 25 to 50 °C with increments of 5 °C. The temperature range was selected based on previous literature studies [47,48,49,50]. The presence of convective currents in the water-based nanofluids will destabilize the rheological properties at higher temperatures. The ternary hybrid nanofluids were loaded within the sample chamber of the rheometer. The double gap spindle was submerged and rotated against the ternary hybrid nanofluid. The drag force acting on the spindle rotation is determined using the calibrated spring’s deflection amplitude. At room temperature, shear strain, shear stress, and nanofluid viscosity were assessed. The data logger on the instrument records the values of the variables being measured. The rheometer is very precise with an accuracy that is guaranteed to be within ±1%. The reproducibility of the spindle speed of the rheometer is within ±0.2%. Ternary hybrid nanofluids have an opposite viscous effect against spindle rotation. Measurements of viscosity, shear stress, and shear strains for ternary hybrid nanofluids are made at different temperatures ranging from 25 to 50 °C with increments of 5 °C for ternary hybrid nanofluids.

## 3. Taguchi Method

Dr. Taguchi invented a system based on orthogonal array (OA) experiments that result in a significantly lower variance for the experiment when control parameters are adjusted to optimal settings. Hence, the design of experiment (DOE) satisfaction with optimizing control parameters to produce BEST findings is accomplished. The OA gives a collection of well-balanced (minimum) trials. Dr. Taguchi’s signal-to-noise ratios (S/N), which are log functions of the desired output, serve as objective functions for optimization and may be used in data analysis and predicting optimal outcomes. The Taguchi method reduces product development time for design and engineering, lowering costs and increasing profits.

The Taguchi method also allows for controlling variances produced by uncontrolled elements that are not considered in the traditional design of experiments. Taguchi measures the performance qualities of the calibers of control factors against these factors by converting the objective function values to a signal-to-noise (S/N) ratio. S/N ratio is defined as the intended signal ratio for the unwanted arbitrary noise value and indicates the quality features of the experimental data. Furthermore, ANOVA is used to assess the statistical significance of the process parameter. The use of ANOVA and S/N ratios ensures that the performance characteristics are accumulated optimally.

### Plan of Experiments

The types of nanomaterials, concentration, temperature, and shear rate were optimized using two levels of Taguchi model, and their effect on viscosity and shear stress was evaluated. The four parameters were allocated values in two levels based on the preliminary study. Table 1 summarizes the variables and their responses.

For viscosity and shear stress, the S/N ratios of four variables were determined. The consequentiality of the factors on viscosity and shear stress was determined using an ANOVA with a 95% confidence level. Minitab 18 software was used to carry out an optimization procedure based on the Taguchi technique. Taguchi’s-OA was used in the design of the experiment for level-2 and parameter-4 combination for eight runs (Table 2).

## 4. Results and Discussion

In general, the rheological behavior of nanofluids is of great importance in a wide range of applications [51]. There is a proportional relationship between viscosity, shear rate, pumping power and pressure decrease, and fluid convective heat transfer. The change in viscosity is primarily due to two significant features, such as temperature change and the increase/decrease in nanoparticle concentrations in the nanofluids. Another factor that increases viscosity is the interaction between k and the viscosity of nanofluids. Many reports have also shown that viscosity is also affected by the element k. However, few studies have demonstrated that viscosity depends on the size of nanomaterials with a variation of more than 5% [52]. Furthermore, the viscosity of nanofluids varies with the material type of nanoparticles and their behavior, whether metallic or non-metallic. Another important aspect is the shape of the particle.

### 4.1. Ternary Hybrid Nanoparticle Type

#### 4.1.1. Effect of Viscosity

**Figure 2:**
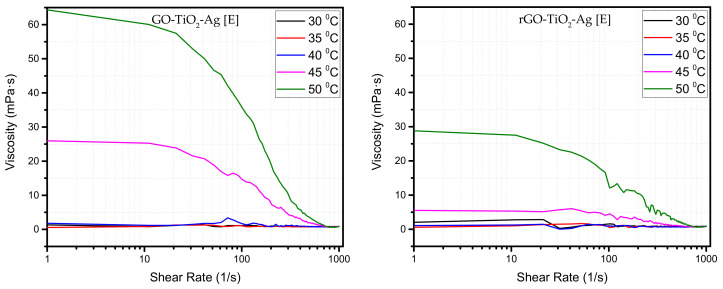
Shear rate vs. viscosity at different temperatures and concentrations of ternary hybrid nanoparticles.

The steady viscosity of nanofluids was evaluated at concentration E (5 × 10^−5^ wt%) by increasing the shear rate from 1 to 1000 L/s concerning temperature. Figure 2 shows that the nanofluids behave like a Newtonian fluid at low temperatures (30 °C, 35 °C, and 40 °C) as the viscosity of nanofluids remained steady with increasing the shear rate; however, at higher temperatures (45 °C and 50 °C), they behave like non-Newtonian fluids with shear-thinning properties, as viscosity has decreased as reported in the previous report [21]. This could be due to long molecule chains of the nanofluids that interact complexly, which results in shear dilution or shear thinning behavior. As the shear rate increases, the attractive forces between the nanoparticles are reduced, and the particles are scattered, reducing viscosity. Shear-thinning could be associated with pseudo-plastic behavior.

This results from minor structural improvements in fluid with microscale fluid geometries to allow the shearing of fluids themselves. Phase separation is another phenomenon that contributes to shear-thinning in nanofluids. Numerous studies have reported the measured rheological properties of different nanofluids. Studies also documented the effects of the presence of nanoparticles on the rheological properties of the base fluid. Rheological studies were carried out to determine whether the decrease in pressure and the structure of nanoparticles can help predict the thermal conductivity of nanofluids [21,53]. Numerous theoretical models for different concentrations have also been presented to date, along with experimental observations of nanofluids [24]. Much of the experiments were performed based on single material nanoparticles, and theoretical models are also derived for the same [54]. Rheological investigations found that viscosity improved compared to the water with nanoparticles [29]. Single component nanoparticles of aluminum oxide, silicon dioxide, and titanium dioxide were used at a concentration of 4.3%. Wang et al. observed pseudo-plastic behavior in the nanofluid with the addition of graphite to oil. Viscosity was improved along with a visco-elastic rise of approximately 1.36 vol% [55]. Newtonian behavior was reported using hybrid nanofluid with Cu-Zn (1:1) alloy with vegetable oil exhibiting a linear relationship between shear stress and shear rate with lower viscosity [56].

#### 4.1.2. Effect of Concentration

**Figure 3:**
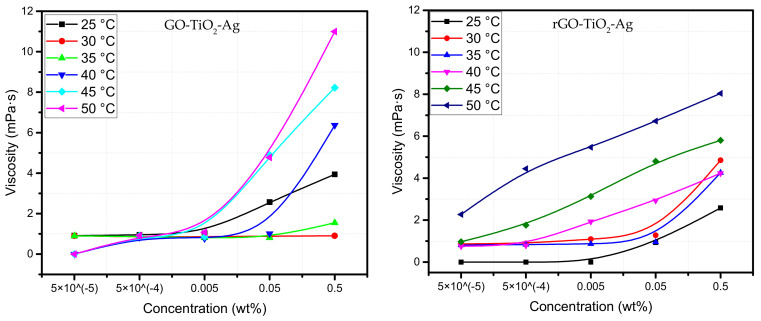
Viscosity vs. concentration at different temperatures of ternary hybrid nanoparticles.

The measurement plots in Figure 3 show the variation of viscosity with the change in the concentration of nanofluids with five levels of serial dilution. The highest concentration is 5 × 10^−1^ wt%, whereas the lowest concentration is 5 × 10^−5^ wt%. The viscosity of two ternary hybrid nanofluids is proportional in terms of their increase in concentration. The viscosity of GO- and rGO-based ternary hybrid nanofluids increases with the increase in concentration at various measured temperatures, albeit at different magnitudes. It can be seen from the plots of both GO- and rGO-based THNPs, the viscosity of individual concentration increases gradually with the increase in temperature. Generally, the viscosity of the water-based nanofluids decreases with the lowering of concentrations of the nanoparticles. From both the plots, it can be seen that at 50 °C, the nanofluids have a spike in viscosity, which can be considered an optimum operating temperature. The GO-based ternary hybrid nanofluid at a concentration of 5 × 10^−1^ wt% at 50 °C increased viscosity by 50% at almost all the temperatures measured. A similar hike in viscosity can be seen in the rGO-based ternary hybrid nanofluids. It can also be noted that GO-based ternary hybrid nanofluid has significantly higher viscosity at all concentrations and temperatures compared to rGO-based ternary hybrid nanofluid. The increased viscosity of GO-based nanofluids could be a result of a variety of causes.

A slight change in the concentration of the nanofluids has the effect of increasing or decreasing the nanofluids’ rheological properties. Almost all studies on nanofluids have concluded the same thing. Substantial efforts have been applied to the research of concentrations or volume fractions of nanoparticles in nanofluids. There is a strong relationship between the increase in the concentration of nanoparticles and the increase in viscosity [56]. Few reports have studied the Newtonian behavior of water-based metallic nanofluids where the viscosity increased with the increase of concentration of nanoparticles on the fluid [29]. The increased viscosity can be as low as 4–15% or as high as 50% [22]. It can be seen that viscosity is detrimental to the increase of thermal conductivity in the nanofluids. However, it cannot be generalized that the increase in nanoparticle concentration will increase viscosity. Figure 3 below shows the viscosity measurements of the two ternary hybrid nanoparticles.

#### 4.1.3. Effect of Temperature

**Figure 4:**
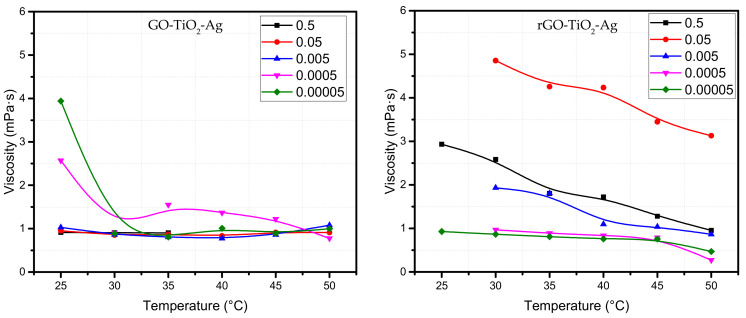
Viscosity vs. temperature at different temperatures and concentrations of ternary hybrid nanoparticles.

The viscosity of water, EG, and oil-based nanofluids decreasing with the fluid’s increase in temperature is a known phenomenon. The rate of decrease in viscosity due to an increase in temperature depends on the fluid’s intrinsic property or the intermolecular bond strength. The increase in temperature of the fluid supplies the molecules in the fluids with higher energy. In the case of nanofluids, which have additional particles, this increase of temperature leads to increased heating and heat transfer leading to faster weakening of the intermolecular forces, which decreases the fluid’s viscosity. The intermolecular interactions between the water-nanoparticle become weaker with the increase in the temperature of the fluid, leading to a decrease of viscosity in the ternary hybrid nanofluid [57]. The strong van der Waals forces act on the particles at lower temperatures. Once the temperature increases, the forces weaken, leading to a decrease in viscosity [58]. Figure 4 depicts the averaged plot of viscosity versus temperature in terms of concentration of GO- and rGO-based ternary hybrid nanofluids. It can be seen that the viscosity is almost similar except for a slight variation, with the lower concentration fluid exhibiting slightly lower viscosity at lower temperatures [59], with the viscosity decreasing to about 80% in GO-based ternary hybrids. At the same time, it is about 33% in the rGO-based THNf. It must be noted that the concentrations of THNp in the nanofluids are very minute (<1 wt%).

The rGO-based ternary hybrid nanofluids show a similar trend in which the viscosity decreases with the increase in temperature of the fluid for all concentrations. The concentration of 0.05 wt% shows a higher viscosity than the other concentration, while the lowest concentration has a lower viscosity. The potential explanation for this phenomenon is that the nanoparticles in fluid aggregates with other particles similar to nanocluster formation due to Van der Waal forces of attraction between nanoparticles and higher resistance to flow as the shear rate is lower at higher temperatures. Other non-covalent forces, such as steric, hydrogen bonding, hydrophobic, and electrostatic attraction, account for the majority of the interactions of the nanofluids with their surroundings [60]. Agglomeration of nanoparticles at higher temperatures creates internal shear stress between particle-fluid interface interactions, increasing viscosity in the hybrid nanofluids. Binding energy is another phenomenon that occurs at higher temperatures resulting in agglomeration or the creation of microclusters in nanofluids which increases viscosity [61]. The hydration layer formation on the nanoparticle-fluid interface increases the cohesion potential between the solid and liquid layers that are typically responsible for transferring or dissipating heat, acting as solid at a nanoscale level. This interaction of solid-liquid layers can be higher at higher temperatures as the liquid layer needs to transfer or dissipate higher amounts of heat energy. This behavior of liquid layers acting as solid particles leads to an increase in viscosity [61]. Nanoparticles form clusters due to higher attractive forces at higher temperatures contributing to increased viscosity [6].

It shows that concentration plays a vital role in the change of viscosity with temperature change. The viscosity increases or decreases with temperature change. The addition of heat changes the nanoparticles in the fluid from surface force attraction to interatomic bonding [62]. The difference is more heightened between different nanofluid concentration samples. This may be due to the agglomeration of nanoparticles at higher temperatures [24]. Similar results were observed by most researchers who studied the effect of temperature on the viscosity of the nanofluids based on different levels of concentration. A viscosity decrease of 56.4% and 73.9% at 50 °C and 70 °C, respectively, was reported in a study on rGO-based ethylene glycol nanofluids [63]. While, other researchers have reported an astronomic viscosity decrease of about 593% at 50 °C for their study of graphite/engine oil nanofluids [59]. Few researchers reported the effect of temperature on the viscosity of hybrid nanofluids. Esfe and Rostamian reported a 171% viscosity decrease from 40 to 100 °C with a volume fraction of 0.05, 0.1, 0.2, 0.4, 0.8, and 1 vol% using CuO-MWCN hybrid nanofluids [25]. When the temperature is increased in all volume fractions of the nanofluids, viscosity decreases exponentially.

#### 4.1.4. Effect of Shear Rate

**Figure 5:**
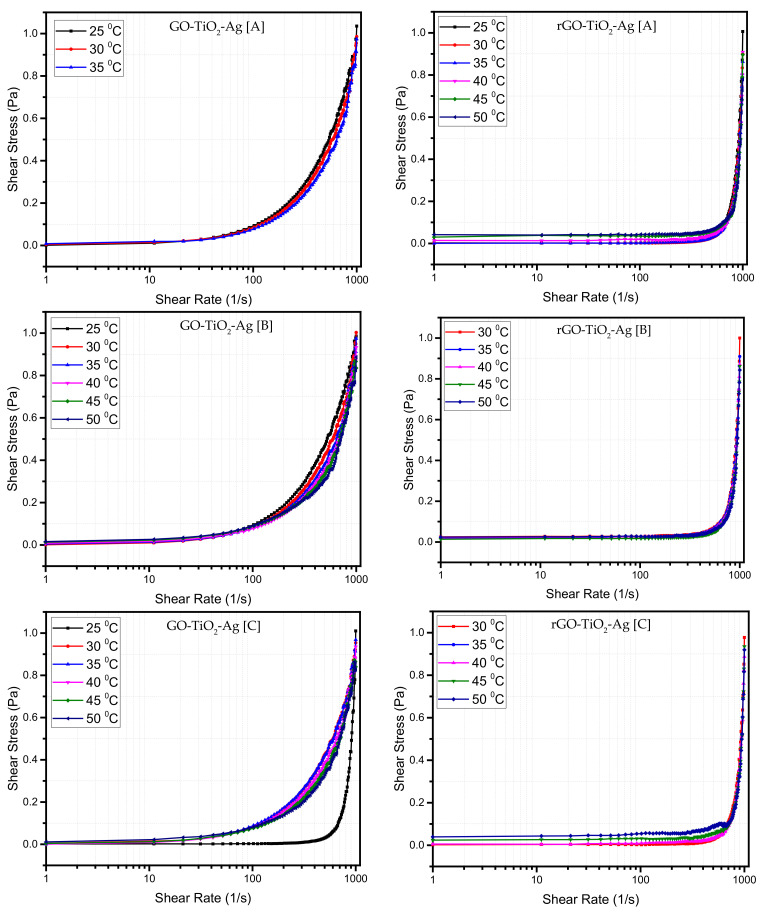

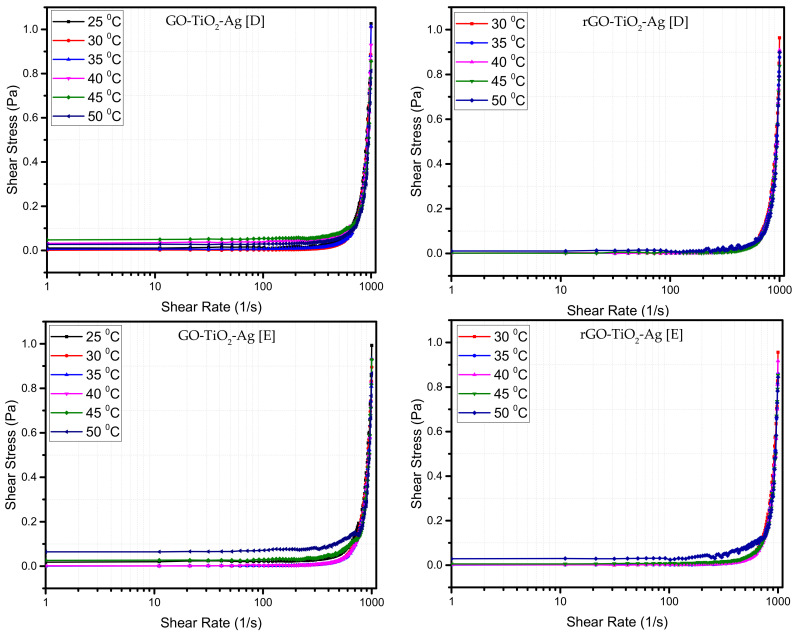
Shear stress vs. shear rate at different temperatures and concentrations of ternary hybrid nanoparticles. (**A**–**E**) are serial dilutions.

Shear stress is the factor that determines whether the fluid is Newtonian or non-Newtonian. The control of the nanofluid suspension’s flow property is crucial in many large-scale industries, such as paint, crude oil drilling, handling and transportation, food processing, and other consumer health products. As mentioned earlier, studying their rheological properties will give them insight into controlling and easy transportation of all manufactured products in the industries. One of the methods to investigate their flow-based property is to determine the shear rate and shear stress of the flow in the fluids. The water-based nanofluids generally behave as shear-thinning fluids at temperatures ranging from 30 to 80 °C [64]. Over the past few decades numerous scholars have studied flow properties. Different characteristics were reported, such as whether the fluids are Newtonian, non-Newtonian or dilatant or pseudo-plastic, or thixotropic fluids. Figure 5 above shows the shear rate versus shear stress in logarithmic scale for the two types of GO-based ternary hybrid nanofluids at various concentrations and temperatures. The plots demonstrate that the viscosity of hybrid nanofluids is almost linear at measured temperatures, suggesting Newtonian fluid. It can also be shown that ternary nanofluids have slightly higher viscosities than base fluids [33].

The GO- and rGO-based ternary hybrid nanofluid behaves as a Newtonian fluid at higher concentration, indicating that shear stress is linearly proportional to the shear strain in the fluids at all the measured temperatures. On the other hand, in the rGO-based ternary hybrid nanofluids, the shear stress is not proportional to the shear strain and behaves as a dilatant or shear thickening fluid at all concentrations. The GO-based ternary hybrid nanofluids gradually start acting as a shear dilatant or shear thickening fluid as the concentration is decreased. The response was seen in the concentration 0.05 wt% (plot B) is known as the structural breakdown of bonds between the molecules with the increase of shear rate. The nanofluid behaves like a thixotropic fluid at a particular concentration and temperature with higher shearing forces. The binding forces or the binding energy are not enough for the solid-liquid layer to retain its structure. It can also be depicted as the breaking or dispersion of the agglomerated particles.

The shear stress and shear strain are a function of concentration and temperature. Studies have revealed that the water-based nanofluids behave as a Newtonian fluid at a lower concentration of nanoparticles in the fluid [65] and as a non-Newtonian fluid at a higher concentration. The nanofluids behaved as shear thickening fluids when the concentration of the nanoparticles decreased [66]. The plots in Figure 5 show the various shear stress versus shear strain at different temperatures. A side-by-side comparison between the GO- and rGO-based ternary hybrid nanofluids is made. The GO-based ternary hybrid nanofluid behaves almost like a Newtonian fluid for sample A at temperatures ranging from 25 to 50 °C, while the rGO-based ternary hybrid nanofluids behave like shear-thickening or dilatant fluids for the same concentration for all temperatures. In the following plot for the second concentration (sample B), the GO-based ternary hybrid nanofluids at lower temperatures of 25 °C behave like Newtonian fluids but start to show slight shear thickening behavior once the temperature is increased till the maximum measured temperature of 50 °C.

In contrast, rGO-based ternary hybrid nanofluids behave as a shear-thinning fluid from 25 °C to 50 °C. The measurements on concentration (sample C) of the GO-based ternary hybrid nanofluids at 25 °C behave like a shear thickening fluid but tend to be a Newtonian fluid once the temperature is increased from 30 °C until the final measured temperature of 50 °C.

There can be many reasons behind the nanofluids’ behavior as Newtonian fluids at higher concentrations and as a dilatant behavior at lower concentrations. At increasing concentrations, the particle-particle interactions become more significant, and the fluid retains its Newtonian properties. Once the shear rate and temperature increase, the particle-to-particle interactions and the attractive forces increase, forcing the nanofluids to behave as a dilatant fluid. On further concentration reduction, the GO-based ternary hybrid nanofluids ultimately act as dilatant or shear thickening fluids. This influence of the concentration of the nanoparticles in the fluid may be due to the higher inter-particle forces at higher concentrations. These higher inter-particle forces influence the nanofluids to remain Newtonian [67]. In contrast, the rGO-based nanofluids remain as shear thickening or dilatant fluids at all concentrations and temperatures. This may be due to the lesser number of oxygen molecules in the rGO nanoparticles. Previous rheological studies on hybrid nanofluids have reported that a higher volume fraction of nanoparticles trigger the formation of larger nano-clusters, increasing the viscosity due to van der Waals forces existing between particles caused by the reduction of movements between fluid layers [68]. As the hydrodynamic diameter of the nanoparticles increases, adsorption and clustering/agglomeration occur, tending to increase the viscosity, as reported in the literature [22]. Agglomeration structures formed in the nanofluids during the shearing process diminish into an orderly arrangement within the shear rate range, as observed by recent research [22]. Studies by Yang et al. on Al_2_O_3_-diamond hybrid on DI water and silicone oil showed that the nanofluids tend to be Newtonian due to agglomeration. However, once the shear rate is increased, it tends to be non-Newtonian shear-thinning fluid similar to the behavior of the GO-based nanofluids in our study. It is also because of the agglomeration due to the dominance of Van der Waals forces at lower shear rates. At higher shear rates, the agglomeration breaks down, reducing viscosity, substantially making the fluid behave as a non-Newtonian shear-thinning fluid.

The plots above show that nanofluids at lower shear rates exhibit Newtonian behavior and non-Newtonian behavior at higher shear rates. Many studies have shown that the temperature is directly related to the change in nanofluids’ rheological properties and thermal conductivity [69]. The rheological measurements on the two GO-based ternary hybrid nanofluids were performed at temperatures ranging from 25 to 50 °C. It can be seen that the shear stress increases with the increase in shear rate and decreases with temperature. The trend in the GO plots shows that at a particular concentration (sample C) and lower temperature (25 °C), the fluid shows dilatant or shear-thinning behavior. However, when the temperature increases (30–50 °C), the nanofluid exhibits Newtonian behavior. On further dilution at a concentration D, the nanofluids ultimately exhibit shear-thinning or pseudo-plastic behavior irrespective of the temperature. The rGO profiles, on the other hand, exhibit shear-thinning or pseudo-plastic behavior at all concentrations and temperatures.

### 4.2. Effect of Amplitude Sweep

The linear viscoelastic (LVE) is a region that indicates the fluid range in which the stresses are tested without destroying the fluid structure. The LVE measurements are another set of rheological measurements that can be determined by measuring stresses divided into storage (G′—elastic) and loss (G″—viscous) moduli. The loss and storage moduli show the mechanical properties of the nanoparticles under low oscillatory shear. It is the range with the lowest strain values. The G′ in the LVE region shows a constant value which is also called the plateau value. These measurements are done using the rheometer in which the spindle performs oscillatory motions of stress range between 0.01 and 10 Pa at a constant frequency of 1 Hz. If G″ > G′, then the fluid can be termed a viscoelastic liquid. When an external strain is applied to the fluids, the structure of the fluid opposes the strains to a certain extent where the loss modulus G′ increases. The fluid structure in LVE then deforms, leading to the dismantling of the aggregations of the nanoparticles in the fluid. This will lead to nanoparticles in the fluids aligning to the flow field, decreasing the storage and loss moduli in the fluid.

**Figure 6:**
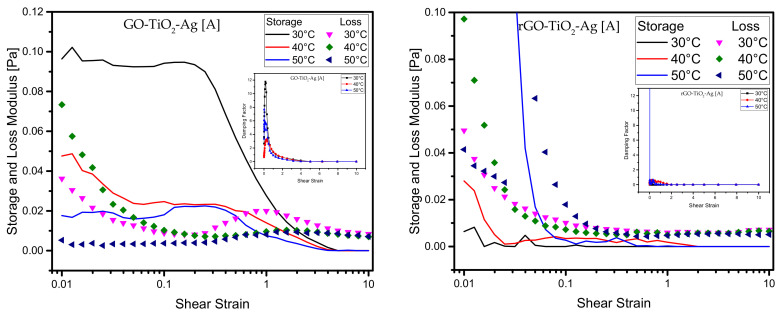

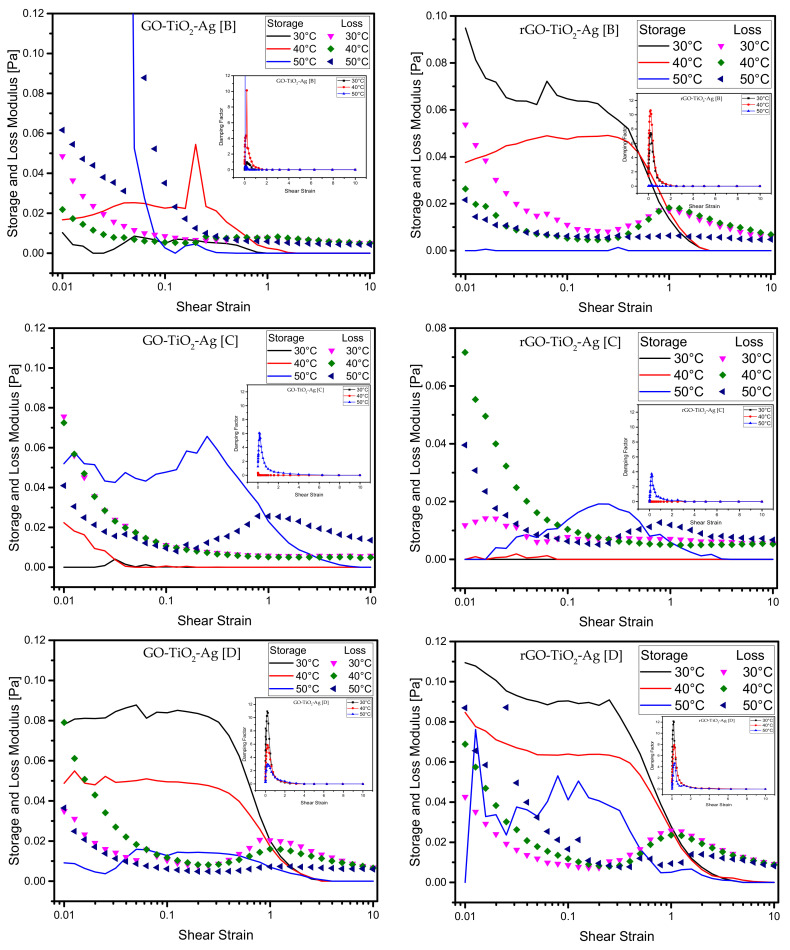

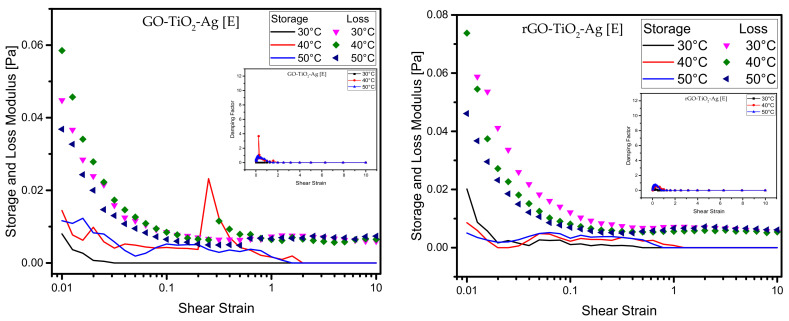
Amplitude sweep—storage modulus and loss modulus vs. shear stress and their damping factor (inset) at different temperatures of ternary hybrid nanoparticles. (**A**–**E**) are serial dilutions.

The plots in Figure 6 show the loss and storage moduli of the two GO-based ternary hybrid nanofluids. It can be seen that the curves demonstrate a similar qualitative feature with a sharp decline of high oscillating stresses and are characterized by constant modulus or linear viscoelastic plateau. It can also be seen from the plots that the loss modulus is higher in the nanofluids with higher concentrations. A crossover area between the loss and storage moduli shows the presence of yield stress in the nanofluids, as mentioned by a few studies. At higher shear strain, the G′ decreases indicating the loss of viscoelastic structure in the nanofluid. One striking feature is that at lower concentrations of both GO- and rGO-based ternary hybrid nanofluids, the loss modulus is almost similar, indicating that the nanofluids lose the ability to store stresses and remain constant at all measured temperatures. This suggests that the nanoparticles in the fluids enhance the rheological capabilities, such as storage, damping, and loss moduli in the fluids.

This could be one of the reasons why heat conductivity and other rheological parameters have been enhanced. According to certain studies, network architectures occur in nanofluids at higher particle concentrations and lower stresses or frequencies [63]. It is noted from the plots that the critical external strain is independent of all concentrations of the fluids. It should be noted that the base fluids exhibit a Newtonian behavior, but the addition of ternary hybrid nanoparticles to the base fluids transitions it to viscoelastic behavior, which is shear-thickening or dilatant in our case. A damping factor is a dimensionless unit that can be described as the ratio of loss over storage moduli (G″/G′) denoted by the term tan δ. The ideal value for the damping factor for a liquid in the viscoelastic region is always greater than unity (tan δ = G″/G′ >1). The damping factor plots are shown in the figure insets in each plot.

### 4.3. Effect of Frequency Sweep

**Figure 7:**
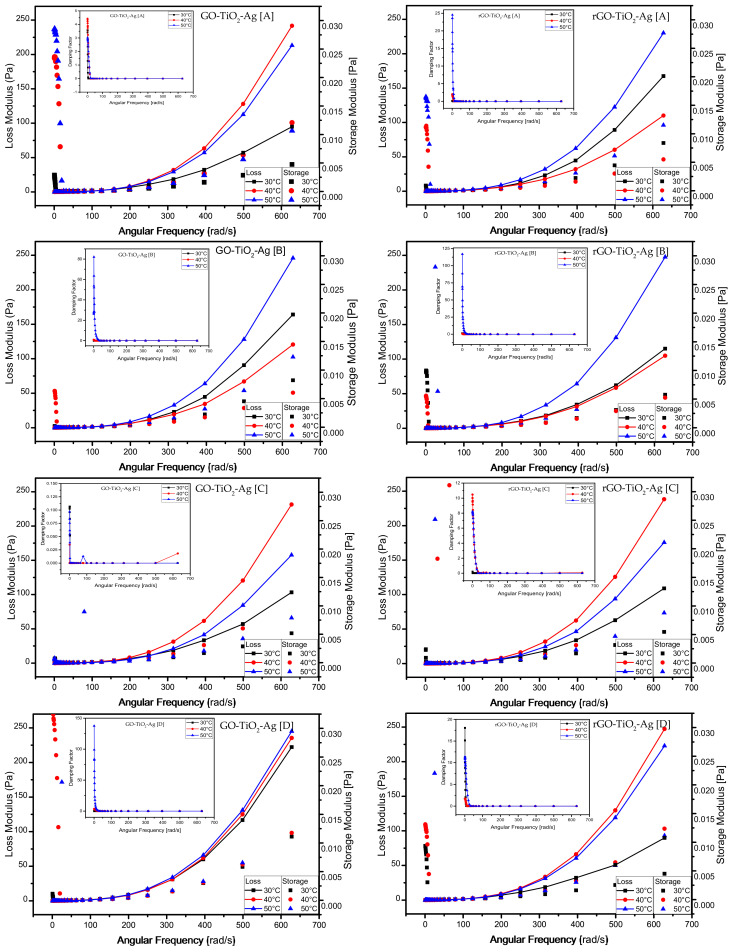

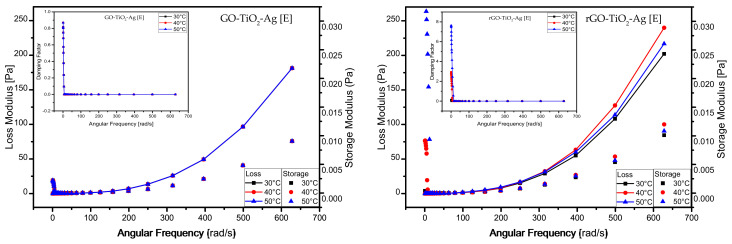
Frequency sweep—storage modulus and loss modulus vs. shear stress and their damping factor (inset) at different temperatures of ternary hybrid nanoparticles. (**A**–**E**) are serial dilutions.

Frequency sweep tests are carried out to measure the time-dependent behavior of the nanofluids in a non-destructive deformation range. It is an essential tool to measure the stability of nanofluids. In addition, it helps to quantify zero shear viscosity for viscoelastic liquids. Frequency sweeps can be measured either by using fast-moving high-frequency simulation or by slow-moving low-frequency simulation. The frequency sweeps were measured in the LVE region with angular frequencies ranging from 0.1 to 10 Hz (0.628 to 62.831 rad/s) by applying oscillatory stress of 0.05 Pa. The loss and storage moduli are similar and practically constant in all the concentrations of the measured ternary hybrid nanofluids. The measurements show that at higher frequencies, both the storage and the loss moduli increase. The damping factor is shown in the Figure 7 insets. It can be seen that the damping factors are higher only in the viscoelastic region with low angular frequency. Once the frequency is increased, the damping factor is minimum and uniform across all the concentrations independent of temperature. Both the GO- and rGO-based ternary hybrid nanofluids exhibit a similar trend for the damping factor. The damping factor decreases exponentially with the increase in angular frequency.

### 4.4. Comparison of Current Experimental Results with Viscosity Models

**Figure 8:**
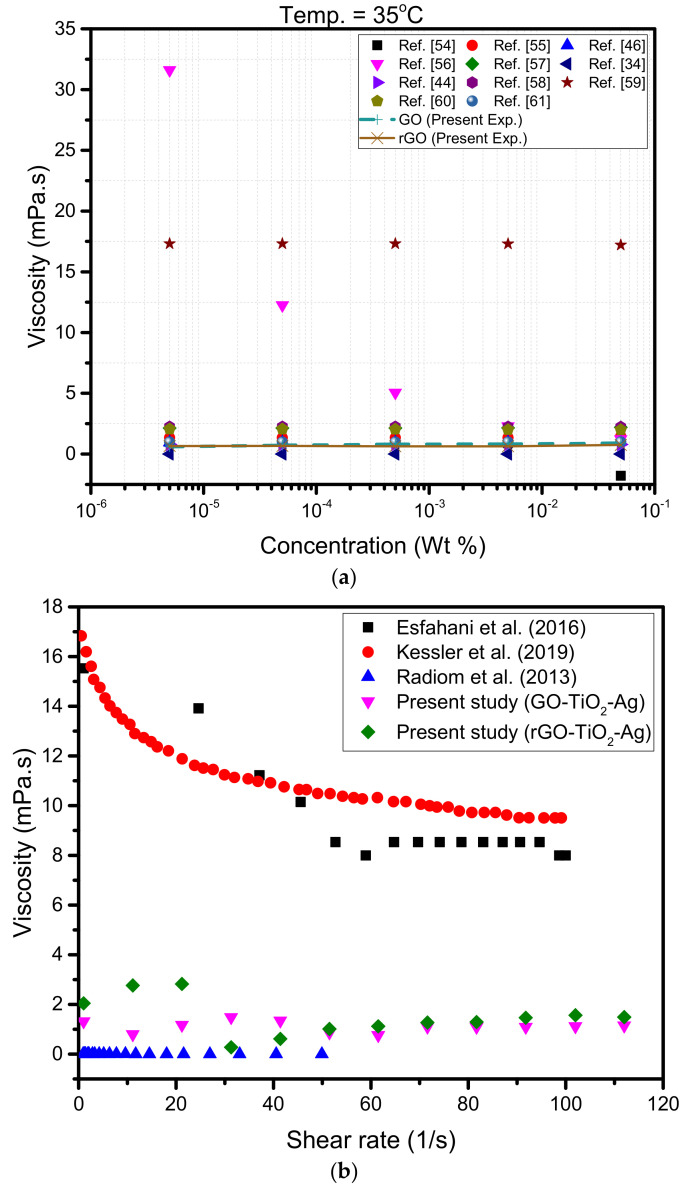
(**a**) Comparison of existing viscosity models with experimental results at 35 °C and (**b**) comparison of experimental viscosity values from literature with current experimental results at 30 °C.

A substantial number of researchers have dwelled on finding the exact cause of the increase of viscosity of hybrid nanofluids. Various studies have developed several correlations for determining the viscosity of hybrid nanoparticles since their inception. Even though they successfully determined the correlations for the nanofluids, studies have not determined a universal correlation for determining viscosity in nanofluids. The correlations obtained thus far have varied according to the type of nanoparticle used, the type of base fluids, the temperature range, the concentration range of nanoparticles, the sonication time of nanoparticles in the base fluids, the presence or absence of surfactant, the number of nanoparticles in a hybrid nanoparticle combination, and so on. The experimental results of this work are compared to a few current hybrid viscosity models of water-based base fluids (Table 3) using a single temperature (35 °C), as shown in Figure 8a. The comparison indicated a few of the hybrid correlations of viscosity and experimental data for ternary hybrid nanofluids. As can be seen from Figure 8, few of the existing water-based hybrid viscosity correlations agree with the experimental results. Figure 8b is the comparison of the experimental results of water-based nanofluids dispersed with individual nanoparticles taken from the literature, such as graphene oxide, silver, and titanium oxide with the ternary hybrid nanoparticles. It can be observed that the viscosity of the ternary hybrid nanofluids are much lower compared to the individual nanoparticle-based nanofluids.

## 5. Taguchi Optimization Results

### 5.1. Analysis of Variance (ANOVA)

The most common method of experimental design for determining the contribution and effects of each specified parameter is an analysis of variance (ANOVA). As a result, the variance analysis for each parameter and a combination of parameters is the first idea to consider. In this study, a two-level OA with eight experimental runs was used. The experiment has a total degree of freedom of 8 − 1 = 7 (Table 4). The interaction impact of each component with all possible combinations of factors is shown in the ANOVA table.

**Figure 9:**
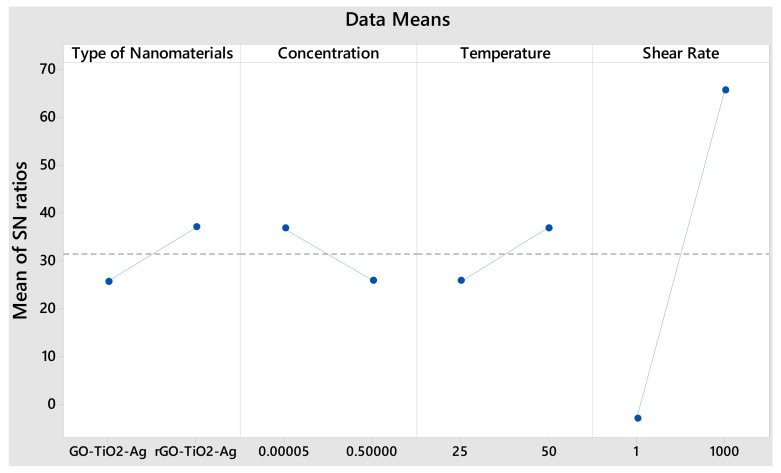
Main effects plot for SN ratios.

The ANOVA investigation analyzes the influence of variables and their associations by comparing the mean square versus response parameters of nanoparticle inaccuracies at set confidence levels. The impact of each component on the total variance of the outcomes may be assessed using ANOVA. Table 4 and Table 5 provide the results of the ANOVA table for all response factor test cases (nanoparticles properties). The study was performed at a significant level of 5%, which equates to 95% trust. Each ANOVA table in the last column shows the proportional contribution of each element’s component to the total variance. Every component contribution that substantially affects a response factor and factors that considerably disrupt output parameters may be assessed using the ANOVA table. Figure 9 supports these findings, and the normalized effect plot is beneficial for screening design. The main effects’ graphs also clearly show the shear rate influence on response variance (Figure 9).

**Figure 10:**
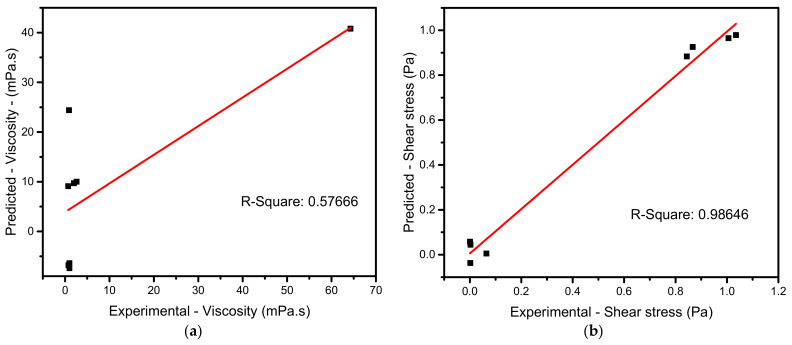
Regression prediction plots. (**a**) Viscosity; (**b**) Shear stress.

According to Table 4, for one-way interactions, the input parameter does not reflect more on response output which has a maximum F-value of 1.11, and it shows the likelihood of being effective. This is because selected parameters are of two kinds: one is a type of nanomaterial, and another is the properties of nanoparticles. Hence, the selection of input parameters should be limited to characterization. The R-square for the response viscosity was found to be significantly less, as shown in Figure 10, which is 0.57666. The provided parameter is statistically significant if the F-value is more than 8%.

Table 5 reveals that shear rate holds the highest significance with F-values of 253.69 on shear stress monitored by an F-value of 0.06 in temperature. In comparison, other parameters’ F-values are not more than that 8%. This shows the shear rate of selected parameters is more influenced by shear stress as it represents the exact mechanical characterization of nanomaterials. A regression model of current parameters shows the F-value of 63.83. This means the shear stress response is statistically suitable for selected inputs.

This is because the 7-DOF used all four components and four interactions, and there was nothing left to enumerate the error. The outcome of non-significant variables and interactions was aggregated as a solution to this problem. There was a slight variation in the encounter. As a result, their DOF and SS have been grouped. 

### 5.2. Coefficient of Factors

The study of factor coefficients is another crucial aspect of optimization. As a result, Table 6 and Table 7 show the factor coefficients as well as their interactions. It can be observed that the concentration has the most significant influence, with a coefficient value of −31.4 (viscosity) and 0.134 (shear stress). However, the other parameter for each reaction varies; for example, temperature follows concentration with 0.642 coefficients for viscosity, but shear rate coefficients are discovered for the shear stress response with 0.000922. When compared to concentration for the response viscosity, the factor coefficient of the shear rate is low and far away, at −0.0165. Temperature is also the bare minimum for shear stress response. Other parameter nanomaterials only reflect the name of materials selected; hence this does not influence the coefficient of factors. The sign of the coefficients is irrelevant since the “weight” of the coefficients is all that matters.

The ANOVA study yielded the following linear regression model, which reflects the output response of two different mechanical characteristics of nanomaterials as a function of concentration, temperature, and shear rate, with an expression of the regression equation as follows.

### 5.3. Data Distribution through a Linear Regression Equation

A statistical model based on linear regression equations was discovered using Taguchi’s orthogonal array and MINITAB software. Each material combination test is represented by a linear regression equation based on each component,

Type of nanomaterial: GO-TiO_2_-Ag

(1) Shear stress=−0.025+0.134×Concentration+0.00058×Temperature+0.000922×Shear Rate

(2) Viscosity=8.8−31.4×Concentration+0.642×Temperature−0.0165×Shear Rate

Type of Nanomaterials: rGO-TiO_2_-Ag

(3) Shear stress=−0.053+0.134×Concentration+0.00058×Temperature+0.000922×Shear Rate

(4) Viscosity=−6.3−31.4×Concentration+0.642×Temperature−0.0165×Shear Rate

The linear regression Equations (1)–(4) were used to calculate the response material test combination values for all test instances to estimate the anticipated outcomes and percentage variance. The residual errors with R-square values of each test are shown in Figure 10 a,b, with the least R-square values being 0.57666 for viscosity and 0.98646 for shear stress. The precision of the operation, the device’s errors in the measuring method, and the combination of nanomaterial characteristics with appropriate formation. Similarly, because process parameters are conditioned for the operations, the experiment is dependent on the number of mechanical characteristics. Expected values are then calculated, actual values deducted, and effects squared. To calculate the total variance, subtract the average absolute value from the actual values, square the results, and count them. The R-square may be used to compare the following values:

(5) $$R^{2}=1-\frac{RSS}{TSS}=1-\sum_{i}\frac{{\left(y_{i}-{\hat{y}}_{i}\right)}^{2}}{{\left(y_{i}-{\bar{y}}_{i}\right)}^{2}}$$

*RSS* stands for “sum of square residuals,” *TSS* for “the total sum of squares,” and *R*^2^ for “coefficient of determination.” Using Equation (5), then divide the second sum (total variance) by the first sum of mistakes (explained variance), subtract the result from one, and obtain the R-square. In this study, the R-square value was found to be a minimum of 57.66%, indicating approximately 43% error in selected parameters for the response viscosity. In contrast, the same combination of parameters is suitable for the shear stress response, and it was found to be 98.64%, indicating statistically appropriateness for optimization study.

## 6. Conclusions

The novel ternary hybrid nanoparticles (GO-TiO_2_-Ag and rGO-TiO_2_-Ag) were synthesized and dispersed at a concentration of 0.5% by weight in double-distilled, deionized water. Afterwards, the nanofluids are diluted sequentially to five different concentrations. Using the zeta potential, the stability of the concentrated stock solution is determined. Measurement results showed that the measured value was steady between 21 and 32 mV. Rheological measurements were performed on an Anton-Paar MCR302 modular compact rheometer. Temperatures ranged from 25 to 50 °C in 5 °C increments for the controlled stress and temperature measurements. GO-TiO_2_-Ag and rGO-TiO_2_-Ag viscosities increase by around 40% and 33%, respectively, when the temperature and shear rate increase.

The findings are as follows:

1. One of the main differences between Newtonian and non-Newtonian shear thickening and dilatation in the GO-based ternary hybrid nanofluids was the concentration at which Newtonian behavior was seen.
2. A possible explanation is that the reduced oxygen molecules are the reason for certain minor differences in viscosity between the two fluids. Reduced nanoparticle concentration causes the viscosity of GO-based nanofluid to increase, whereas rGO-based nanofluid viscosity decreases.
3. The temperature-dependent variation in viscosity is influenced by concentration. At lower temperatures, particles are subject to Van der Waals forces, but there is only a mild attraction force at higher temperatures, which reduces viscosity. Higher temperatures necessitate agglomeration, which is critical. Other non-covalent forces, such as steric, hydrogen bonding, hydrophobic, and electrostatic attraction, account for the majority of the interactions of the nanofluids with their surroundings.
4. The GO-based nanofluid behaves as a Newtonian fluid at higher concentrations, whereas the rGO behaves as a dilatant fluid or shear thickening. At lower concentrations, GO-based nanofluids exhibit similar tendencies to rGO-based ternary hybrid nanofluids.
5. The linear viscoelastic (LVE) zone is present in the non-linear visco-elastic fluids under the influence of concentration, temperature, and stresses. Oscillating angle sweep tests and frequency sweep tests were used to undertake non-destructive stress evaluations—the loss modulus of nanofluids increases as the concentration increases. Plots of the loss or damping factor ratio revealed that the loss factor is higher exclusively in the viscoelastic zone with low angular frequency. However, at higher angular frequencies, it is at its minimum and constant regardless of temperature.
6. As a final step in the data optimization process, the Taguchi method was used to show how input factors influence output responses by adjusting their levels and interactions. With an R-square value of 0.57666, it was discovered that the current input parameter and its interaction did not affect the viscosity output. With the same input, the shear stress response was perfectly stable.

## Figures and Tables

**Table 1:** Selected parameters and levels.

P	L1	L3
Type of Nanomaterials	GO-TiO_2_-Ag	rGO-TiO_2_-Ag
Concentration	0.5	0.00005
Temperature	25	50
Shear Rate	1	1000

**Table 2:** Orthogonal Array P-4 and L-3.

Run	Parameters				Response	
	Type of Nanomaterials	Concentration	Temperature	Shear Rate	Viscosity	Shear Stress
Unit	-	wt%	°C	1/s	mPa·s	Pa
1	GO-TiO_2_-Ag	0.5	25	1	0.7	0.000738
2	GO-TiO_2_-Ag	0.5	25	1000	1	1.0352
3	GO-TiO_2_-Ag	0.00005	50	1	64.324	0.064335
4	GO-TiO_2_-Ag	0.00005	50	1000	0.86646	0.86665
5	rGO-TiO_2_-Ag	0.5	50	1	2.5824	0.002582
6	rGO-TiO_2_-Ag	0.5	50	1000	1.0062	1.0064
7	rGO-TiO_2_-Ag	0.00005	25	1	2.0453	0.002046
8	rGO-TiO_2_-Ag	0.00005	25	1000	0.84368	0.84386

**Table 3:** Viscosity models for water and ethylene glycol-based hybrid nanofluids.

Hybrid Nanoparticles/Base Fluid	Correlations	Temperature °C	Concentration vol%	Author
Ag–MgO/water	$\frac{\mu_{nf}}{\mu_{bf}}=1+32.795\varphi -7214\varphi^{2}-714600\varphi^{3}-0.11941\times 10^{8}\varphi^{4}$	-	0 < ∅ < 0.02	[70]
ND–Fe_3_O_4_/water ND–Fe_3_O_4_/EG–water (20:80, 40:60 and 60:40)	$\frac{\mu_{nf}}{\mu_{bf}}=ae^{b\varphi }$	T = 20: a = 1.444; b = 1.402; T = 30: a = 1.368; b = 1.472; T = 40: a = 1.277; b = 1.625; T = 50: a = 1.288; b = 1.771; T = 60: a = 1.338; b = 1.655	0 < ∅ < 0.002	[71]
ND–Co_3_O_4_/water ND–Co_3_O_4_/EG ND–Co_3_O_4_/EG–water (20:80, 40:60 and 60:40)	$\frac{\mu_{nf}}{\mu_{bf}}=0.9595{\left(1+\varphi \right)}^{2.399}$	20 < T < 60	0.0005 < ∅ < 0.0015	[61]
MgO–MWCNT/EG	$\frac{\mu_{hnf}}{\mu_{bf}}=\left[0.191\varphi +0.240\left(T^{-0.342}\varphi^{-0.473}\right)\right]\exp\left[1.45T^{0.120}\varphi^{0.158}\right]$	30 < T < 60	0 < ∅ < 1	[72]
MgO–MWCNTs/water–EG	$\mu_{hnf}=3.371\times e^{0.5782\varphi -0.000371T^{2}}$	25 < T < 60	0.025 < ∅ < 0.8	[73]
Fe_2_O_3_–MWCNTs/EG	$\frac{\mu_{hnf}}{\mu_{bf}}=\frac{-2.0987+{\left(4.65\varphi \right)}^{0.0969}+{\left(0.8702T\right)}^{0.2633}+\left(62323.1365\varphi^{2}\right)}{\left(143.1076T^{2}\right)}$	25 < T < 50	0.8 < ∅ < 1.8	[47]
TiO_2_–SiO_2_/water, EG	$\frac{\mu_{hnf}}{\mu_{bf}}=37{\left(0.1+\frac{\varphi }{100}\right)}^{1.59}{\left(0.1+\frac{T}{80}\right)}^{0.31}$	30 < T < 70	0.5 < ∅ < 3	[58]
TiO_2_–SiO_2_/water, EG	$\frac{\mu_{hnf}}{\mu_{bf}}=1.42+{\left(1+R\right)}^{-0.3063}{\left(\frac{T}{80}\right)}^{0.2321}$ *R* is the fraction of particle in mixture	30 < T < 80	(1 vol%) 20:80, 40:60, 50:50, 60:40, and 80:20	[74]
Nanodiamond–Co_3_O_4_/Eg (40:60)	$\frac{\mu_{hnf}}{\mu_{bf}}=0.50437+4.38836\varphi -0.04183T-0.26696\varphi T-22.66087\varphi^{2}+0.00121T^{2}\;+0.003325\varphi^{2}T+0.00332T^{2}\varphi +0.00001T^{3}$	20 < T < 60	0.5 < ∅ < 1.15	[75]
Al_2_O_3_–TiO_2_/water	$\frac{\mu_{nf}}{\mu_{bf}}=2.06-1.32\varphi_{np1}-0.96\varphi_{np2}+0.58\varphi^{2}{}_{np1}-0.39\varphi^{2}{}_{np2}+1.89\varphi_{np1}\varphi_{np2}$	25 < T < 25	1 < ∅ < 2	[76]
SiO_2_–graphite/water	$\frac{\mu_{hnf}}{\mu_{bf}}=1.00527\times \left(T^{0.00035}\right){\left(1+\varphi \right)}^{9.36265}{\left(\frac{W_{G}}{W_{sio2}}\right)}^{-0.028935}$	15 < T < 60	0.1 < ∅ < 2	[77]
(SiO_2_–CuO/C)/glycol–EG	$\frac{\mu_{hnf}}{\mu_{bf}}=0.9894{\left[1+\frac{\varphi }{100}\right]}^{6.6301}\times {\left[\frac{T_{nf}}{T_{o}}\right]}^{0.064}$	50 < T < 80	0.05 < ∅ < 1	[78]

**Table 4:** Analysis of variance (viscosity).

Source	DF	Adj SS	Adj MS	*F*-Value	*p*-Value
Regression	4	2007.5	501.9	1.02	0.513
Concentration	1	492.8	492.8	1.00	0.390
Temperature	1	515.0	515.0	1.05	0.381
Shear Rate	1	543.4	543.4	1.11	0.370
Type of Nanomaterials	1	456.2	456.2	0.93	0.406
Error	3	1472.0	490.7	-	-
Total	7	3479.5	-	-	-

**Table 5:** Analysis of variance (shear stress).

Source	DF	Adj SS	Adj MS	*F*-Value	*p*-Value
Regression	4	1.70599	0.42650	63.83	0.003
Concentration	1	0.00898	0.00898	1.34	0.330
Temperature	1	0.00042	0.00042	0.06	0.818
Shear Rate	1	1.69502	1.69502	253.69	0.001
Type of Nanomaterials	1	0.00157	0.00157	0.23	0.661
Error	3	0.02004	0.00668	-	-
Total	7	1.72603	-	-	-

**Table 6:** Coefficient table (viscosity).

Term	Coef	SE Coef	*T*-Value	*p*-Value	VIF
Constant	8.8	28.2	0.31	0.777	
Concentration	−31.4	31.3	−1.00	0.390	1.00
Temperature	0.642	0.627	1.02	0.381	1.00
Shear Rate	−0.0165	0.0157	−1.05	0.370	1.00
**Type of Nanomaterials**					
GO-TiO_2_-Ag	0.000000	0.000000	-	-	-
rGO-TiO_2_-Ag	−15.1	15.7	−0.96	0.406	1.00

**Table 7:** Coefficient table (shear rate).

Term	Coef	SE Coef	*T*-Value	*p*-Value	VIF
Constant	−0.025	0.104	−0.24	0.827	
Concentration	0.134	0.116	1.16	0.330	1.00
Temperature	0.00058	0.00231	0.25	0.818	1.00
Shear Rate	0.000922	0.000058	15.93	0.001	1.00
**Type of Nanomaterials**					
GO-TiO_2_-Ag	0.000000	0.000000	-	-	-
rGO-TiO_2_-Ag	−0.0280	0.0578	−0.48	0.661	1.00

## Data Availability

Raw data available from the corresponding authors upon request.

## References

[1] Lee S., Choi S.U.-S., Li S., Eastman J. (1999). Measuring Thermal Conductivity of Fluids Containing Oxide Nanoparticles. J. Heat Transf..

[2] Chakraborty S., Panigrahi P.K. (2020). Stability of nanofluid: A review. Appl. Therm. Eng..

[3] Yang L., Ji W., Mao M., Huang J.N. (2020). An updated review on the properties, fabrication and application of hybrid-nanofluids along with their environmental effects. J. Clean. Prod..

[4] Sureshkumar R., Mohideen S.T., Nethaji N. (2013). Heat transfer characteristics of nanofluids in heat pipes: A review. Renew. Sustain. Energy Rev..

[5] Gupta M., Singh V., Kumar R., Said Z. (2017). A review on thermophysical properties of nanofluids and heat transfer applications. Renew. Sustain. Energy Rev..

[6] Babar H., Sajid M.U., Ali H.M. (2019). Viscosity of hybrid nanofluids: A critical review. Therm. Sci..

[7] Rasheed A.K., Khalid M., Rashmi W., Gupta T., Chan A. (2016). Graphene based nanofluids and nanolubricants—Review of recent developments. Renew. Sustain. Energy Rev..

[8] Mekheimer K.S., Hasona W.M., Abo-Elkhair R.E., Zaher A.Z. (2018). Peristaltic blood flow with gold nanoparticles as a third grade nanofluid in catheter: Application of cancer therapy. Phys. Lett. A.

[9] Babar H., Ali H.M. (2019). Towards hybrid nanofluids: Preparation, thermophysical properties, applications, and challenges. J. Mol. Liq..

[10] Khaliq A., Kafafy R., Salleh H.M., Faris W.F. (2012). Enhancing the efficiency of polymerase chain reaction using graphene nanoflakes. Nanotechnology.

[11] Khaliq A., Sonawane P.J., Sasi B.K., Sahu B.S., Pradeep T., Das S.K., Mahapatra N.R. (2010). Enhancement in the efficiency of polymerase chain reaction by TiO_2_ nanoparticles: Crucial role of enhanced thermal conductivity. Nanotechnology.

[12] Sharma D., Pandey K., Debbarma A., Choubey G. (2017). Numerical Investigation of heat transfer enhancement of SiO_2_-water based nanofluids in Light water nuclear reactor. Mater. Today Proc..

[13] Zakaria I., Azmi W.H., Mohamed W.A.N.W., Mamat R., Najafi G. (2015). Experimental Investigation of Thermal Conductivity and Electrical Conductivity of Al_2_O_3_ Nanofluid in Water—Ethylene Glycol Mixture for Proton Exchange Membrane Fuel Cell Application. Int. Commun. Heat Mass Transf..

[14] Sidik N.A.C., Jamil M.M., Japar W.M.A.A., Adamu I.M. (2017). A review on preparation methods, stability and applications of hybrid nanofluids. Renew. Sustain. Energy Rev..

[15] Devendiran D.K., Amirtham V.A. (2016). A review on preparation, characterization, properties and applications of nanofluids. Renew. Sustain. Energy Rev..

[16] Arshad A., Jabbal M., Yan Y., Reay D. (2019). A review on graphene based nanofluids: Preparation, characterization and applications. J. Mol. Liq..

[17] Azmi W., Sharma K., Mamat R., Najafi G., Mohamad M. (2016). The enhancement of effective thermal conductivity and effective dynamic viscosity of nanofluids—A review. Renew. Sustain. Energy Rev..

[18] Aybar H.Ş., Sharifpur M., Azizian M.R., Mehrabi M., Meyer J.P. (2015). A Review of Thermal Conductivity Models for Nanofluids. Heat Transf. Eng..

[19] Pinto R.V., Fiorelli F.A.S. (2016). Review of the mechanisms responsible for heat transfer enhancement using nanofluids. Appl. Therm. Eng..

[20] Esfahani M.R., Languri E.M., Nunna M.R. (2016). Effect of particle size and viscosity on thermal conductivity enhancement of graphene oxide nanofluid. Int. Commun. Heat Mass Transf..

[21] Sharma A.K., Tiwari A.K., Dixit A.R. (2016). Rheological behaviour of nanofluids: A review. Renew. Sustain. Energy Rev..

[22] Podskoczy A. (2016). Beer reigns among holiday drinks, National Social-Political and Economic-Legal Journal Rzeczpospolita. Renew. Sustain. Energ. Rev..

[23] Meyer J.P., Adio S.A., Sharifpur M., Nwosu P.N. (2016). The Viscosity of Nanofluids: A Review of the Theoretical, Empirical, and Numerical Models. Heat Transf. Eng..

[24] Koca H.D., Doganay S., Turgut A., Tavman I.H., Saidur R., Mahbubul I.M. (2018). Effect of particle size on the viscosity of nanofluids: A review. Renew. Sustain. Energy Rev..

[25] Esfe M.H., Zabihi F., Rostamian H., Esfandeh S. (2018). Experimental investigation and model development of the non-Newtonian behavior of CuO-MWCNT-10w40 hybrid nano-lubricant for lubrication purposes. J. Mol. Liq..

[26] Kumar V., Sarkar J. (2019). Numerical and experimental investigations on heat transfer and pressure drop characteristics of Al_2_O_3_-TiO_2_ hybrid nanofluid in minichannel heat sink with different mixture ratio. Powder Technol..

[27] Amini F., Miry S.Z., Karimi A., Ashjaee M. (2019). Experimental Investigation of Thermal Conductivity and Viscosity of SiO_2_/Multiwalled Carbon Nanotube Hybrid Nanofluids. J. Nanosci. Nanotechnol..

[28] Hussien A.A., Abdullah M.Z., Yusop N.M., Al-Nimr M.A., Atieh M.A., Mehrali M. (2017). Experiment on forced convective heat transfer enhancement using MWCNTs/GNPs hybrid nanofluid and mini-tube. Int. J. Heat Mass Transf..

[29] Khadanga V., Rao K., Ram Vikas K., Ram N.R. (2019). Rheological Behaviour of Nano Fluid. Int. J. Res. Anal. Rev..

[30] Bhatia E., Banerjee R. (2020). Hybrid silver-gold nanoparticles suppress drug resistant polymicrobial biofilm formation and intracellular infection. J. Mater. Chem. B.

[31] Perera S.D., Mariano R.G., Vu K., Nour N., Seitz O., Chabal Y., Balkus K.J. (2012). Hydrothermal synthesis of graphene-TiO_2_ nanotube composites with enhanced photocatalytic activity. ACS Catal..

[32] Routbort J.L., Singh D., Timofeeva E.V., Yu W., France D.M. (2011). Pumping power of nanofluids in a flowing system. J. Nanopart. Res..

[33] Ahmadi M.H., Mohseni-Gharyehsafa B., Farzaneh-Gord M., Jilte R., Kumar R., Chau K.-W. (2019). Applicability of connectionist methods to predict dynamic viscosity of silver/water nanofluid by using ANN-MLP, MARS and MPR algorithms. Eng. Appl. Comput. Fluid Mech..

[34] Selvakumar P. (2021). Enabling Taguchi method with grey relational analysis to optimize the parameters of TiO_2_/ZnO heat transfer nanofluid for heat pipe application. Nano Express.

[35] Elcioglu E.B., Yazicioglu A.G., Turgut A., Anagun A.S. (2018). Experimental study and Taguchi Analysis on alumina-water nanofluid viscosity. Appl. Therm. Eng..

[36] Nikhil M.A.N.E., HEMADRİ V. (2021). Study of the effect of preparation parameters on thermal conductivity of metal oxide nanofluids using Taguchi method. J. Energy Syst..

[37] Kumar H., Harsha A. (2021). Taguchi optimization of various parameters for tribological performance of polyalphaolefins based nanolubricants. Proc. Inst. Mech. Eng. Part J J. Eng. Tribol..

[38] Verma T.N., Rajak U., Dasore A., Afzal A., Manokar A.M., Aabid A., Baig M. (2021). Experimental and empirical investigation of a CI engine fuelled with blends of diesel and roselle biodiesel. Sci. Rep..

[39] Sharath B.N., Venkatesh C.V., Afzal A., Aslfattahi N., Aabid A., Baig M., Saleh B. (2021). Multi Ceramic Particles Inclusion in the Aluminium Matrix and Wear Characterization through Experimental and Response. Materials.

[40] Aabid A., Hrairi M., Ali J.S.M. (2020). Optimization of composite patch repair for center-cracked rectangular plate using design of experiments method. Mater. Today Proc..

[41] Aabid A., Khan S.A. (2021). Investigation of High-Speed Flow Control from CD Nozzle Using Design of Experiments and CFD Methods. Arab. J. Sci. Eng..

[42] Al-Khalifah T., Aabid A., Khan S.A., Bin Azami M.H., Baig M. (2021). Response surface analysis of nozzle parameters at supersonic flow through microjets. Aust. J. Mech. Eng..

[43] Afzal A., Aabid A., Khan A., Khan S.A., Rajak U., Verma T.N., Kumar R. (2020). Response surface analysis, clustering, and random forest regression of pressure in suddenly expanded high-speed aerodynamic flows. Aerosp. Sci. Technol..

[44] Sidik N.A.C., Adamu I.M., Jamil M.M. (2020). Preparation Methods and Thermal Performance of Hybrid Nanofluids. J. Adv. Res. Appl. Mech..

[45] Zayan M., Rasheed A.K., John A., Muniandi S., Fen Leo B., Faris A. (2021). Synthesis and Characterization of Novel Ternary Hybrid Nanoparticles as Thermal Additives in H_2_O. ChemRxiv.

[46] Ahmadi M.H., Mohseni-Gharyehsafa B., Ghazvini M., Goodarzi M., Jilte R.D., Kumar R. (2020). Comparing various machine learning approaches in modeling the dynamic viscosity of CuO/water nanofluid. J. Therm. Anal. Calorim..

[47] Nadooshan A.A., Eshgarf H., Afrand M. (2018). Measuring the viscosity of Fe_3_O_4_-MWCNTs/EG hybrid nanofluid for evaluation of thermal efficiency: Newtonian and non-Newtonian behavior. J. Mol. Liq..

[48] Esfahani N.N., Toghraie D., Afrand M. (2018). A new correlation for predicting the thermal conductivity of ZnO–Ag (50%–50%)/water hybrid nanofluid: An experimental study. Powder Technol..

[49] Huminic G., Huminic A. (2018). Hybrid nanofluids for heat transfer applications—A state-of-the-art review. Int. J. Heat Mass Transf..

[50] Huminic G., Huminic A., Dumitrache F., Fleacă C., Morjan I. (2020). Study of the thermal conductivity of hybrid nanofluids: Recent research and experimental study. Powder Technol..

[51] Sidik N.A.C., Adamu I.M., Jamil M.M., Kefayati G., Mamat R., Najafi G. (2016). Recent progress on hybrid nanofluids in heat transfer applications: A comprehensive review. Int. Commun. Heat Mass Transf..

[52] Hamzah M.H., Sidik N.A.C., Ken T.L., Mamat R., Najafi G. (2017). Factors affecting the performance of hybrid nanofluids: A comprehensive review. Int. J. Heat Mass Transf..

[53] Ahmadi M.H., Mirlohi A., Nazari M.A., Ghasempour R. (2018). A review of thermal conductivity of various nanofluids. J. Mol. Liq..

[54] Babu J.R., Kumar K.K., Rao S.S. (2017). State-of-art review on hybrid nanofluids. Renew. Sustain. Energy Rev..

[55] Wang B., Wang X., Lou W., Hao J. (2012). Thermal conductivity and rheological properties of graphite/oil nanofluids. Colloids Surf. A Physicochem. Eng. Asp..

[56] Kumar M.S., Vasu V., Gopal A.V. (2016). Thermal conductivity and rheological studies for Cu–Zn hybrid nanofluids with various basefluids. J. Taiwan Inst. Chem. Eng..

[57] Esfe M.H., Esfandeh S. (2020). A new generation of hybrid-nanofluid: Thermal properties enriched lubricant fluids with controlled viscosity amount. SN Appl. Sci..

[58] Nabil M., Azmi W., Hamid K.A., Mamat R., Hagos F.Y. (2017). An experimental study on the thermal conductivity and dynamic viscosity of TiO_2_-SiO_2_ nanofluids in water: Ethylene glycol mixture. Int. Commun. Heat Mass Transf..

[59] Hu X., Yin D., Xie J., Chen X., Bai C. (2020). Experimental study of viscosity characteristics of graphite/engine oil (5 W-40) nanofluids. Appl. Nanosci..

[60] Doane T., Burda C. (2013). Nanoparticle mediated non-covalent drug delivery. Adv. Drug Deliv. Rev..

[61] Sundar L.S., Irurueta G., Ramana E.V., Singh M.K., Sousa A. (2016). Thermal conductivity and viscosity of hybrid nanfluids prepared with magnetic nanodiamond-cobalt oxide (ND-Co_3_O_4_) nanocomposite. Case Stud. Therm. Eng..

[62] Ali N., Teixeira J.A., Addali A. (2018). A Review on Nanofluids: Fabrication, Stability, and Thermophysical Properties. J. Nanomater..

[63] Shah S.N.A., Shahabuddin S., Sabri M.F.M., Salleh M.F.M., Ali M.A., Hayat N., Sidik N.A.C., Samykano M., Saidur R. (2020). Experimental investigation on stability, thermal conductivity and rheological properties of rGO/ethylene glycol based nanofluids. Int. J. Heat Mass Transf..

[64] HYang H., Yao G., Wen D. (2019). Experimental investigation on convective heat transfer of Shear-thinning fluids by elastic turbulence in a serpentine channel. Exp. Therm. Fluid Sci..

[65] Mahbubul I.M., Saidur R., Amalina M.A. (2012). Latest developments on the viscosity of nanofluids. Int. J. Heat Mass Transf..

[66] Halelfadl S., Estellé P., Aladag B., Doner N., Maré T. (2013). Viscosity of carbon nanotubes water-based nanofluids: Influence of concentration and temperature. Int. J. Therm. Sci..

[67] Kaggwa A., Carson J.K. (2019). Developments and future insights of using nanofluids for heat transfer enhancements in thermal systems: A review of recent literature. Int. Nano Lett..

[68] Afrand M., Najafabadi K.N., Akbari M. (2016). Effects of temperature and solid volume fraction on viscosity of SiO_2_-MWCNTs/SAE40 hybrid nanofluid as a coolant and lubricant in heat engines. Appl. Therm. Eng..

[69] Colangelo G., Favale E., Miglietta P., Milanese M., de Risi A. (2016). Thermal conductivity, viscosity and stability of Al_2_O_3_-diathermic oil nanofluids for solar energy systems. Energy.

[70] Esfe M.H., Arani A.A.A., Rezaie M., Yan W.M., Karimipour A. (2015). Experimental determination of thermal conductivity and dynamic viscosity of Ag-MgO/water hybrid nanofluid. Int. Commun. Heat Mass Transf..

[71] Sundar L.S., Singh M.K., Sousa A.C.M. (2016). Experimental thermal conductivity and viscosity of nanodiamond-based propylene glycol and water mixtures. Diam. Relat. Mater..

[72] Soltani O., Akbari M. (2016). Effects of temperature and particles concentration on the dynamic viscosity of MgO-MWCNT/ethylene glycol hybrid nanofluid: Experimental study. Phys. E Low-Dimens. Syst. Nanostruct..

[73] Zareie A., Akbari M. (2017). Hybrid nanoparticles effects on rheological behavior of water-EG coolant under different temperatures: An experimental study. J. Mol. Liq..

[74] Hamid K.A., Azmi W.H., Nabil M.F., Mamat R., Sharma K.V. (2018). Experimental investigation of thermal conductivity and dynamic viscosity on nanoparticle mixture ratios of TiO_2_-SiO_2_ nanofluids. Int. J. Heat Mass Transf..

[75] Esfe M.H., Hajmohammad M.H. (2017). Thermal conductivity and viscosity optimization of nanodiamond-Co_3_O_4_/EG (40:60) aqueous nanofluid using NSGA-II coupled with RSM. J. Mol. Liq..

[76] Moldoveanu G.M., Minea A.A., Iacob M., Ibanescu C., Danu M. (2018). Experimental study on viscosity of stabilized Al_2_O_3_, TiO_2_ nanofluids and their hybrid. Thermochim. Acta.

[77] Dalkılıç A.S., Açıkgöz Ö., Küçükyıldırım B.O., Eker A.A., Lüleci B., Jumpholkul C., Wongwises S. (2018). Experimental investigation on the viscosity characteristics of water based SiO_2_-graphite hybrid nanofluids. Int. Commun. Heat Mass Transf..

[78] Akilu S., Baheta A.T., M. Said M.A., Minea A.A., Sharma K. (2018). Properties of glycerol and ethylene glycol mixture based SiO_2_-CuO/C hybrid nanofluid for enhanced solar energy transport. Sol. Energy Mater. Sol. Cells.

